# The effects of cognitive leisure activities on frailty transitions in older adults in China: a CHARLS-Based longitudinal study

**DOI:** 10.1186/s12889-024-18889-w

**Published:** 2024-05-27

**Authors:** Kai Sheng, Hao Chen, Xianguo Qu

**Affiliations:** 1 The Fourth School of Clinical Medicine, Zhejiang Chinese Medical University, Hangzhou First People’s Hospital, Hangzhou, China; 2 Affiliated Hangzhou First People’s Hospital, School of Medicine, Westlake University, Hangzhou, China; 3 School of Medicine, Tongji University, Shanghai, China; 4 School of Public Health, Fudan University, Shanghai, China

**Keywords:** Frailty, Transition, Cognitive leisure activities, CHARLS

## Abstract

**Background:**

In an effort to identify factors associated with frailty transitions that trigger a significant difference in preventing and postponing the progression of frailty, questions regarding the role of cognitive leisure activities on various aspects of older adults’ health were raised. However, the relationship between cognitive leisure activities and frailty transitions has rarely been studied.

**Methods:**

A total of 5367 older Chinese adults aged over 60 years from the China Health and Retirement Longitudinal Study (CHARLS) were selected as participants. The 2nd wave of the CHARLS in 2013 was selected as the baseline, and sociodemographic and health-related status baseline data were collected. The FRAIL Scale was used to measure frailty, while cognitive leisure activities were measured by the Cognitive Leisure Activity Index (CLAI) scores, which consisted of playing mahjong or cards, stock investment, and using the internet. After two years of follow-up, frailty transition from baseline was assessed at the 3rd wave of the CHARLS in 2015. Ordinal logistic regression analysis was used to examine the relationship between cognitive leisure activities and frailty transitions.

**Results:**

During the two-year follow-up of 5367 participants, the prevalence of frailty that improved, remained the same and worsened was 17.8% (957/5367), 57.5% (3084/5367) and 24.7% (1326/5367), respectively. Among all participants, 79.7% (4276/5367), 19.6% (1054/5367), and 0.7% (37/5367) had CLAI scores of 0, 1, and 2 to 3, respectively. In the univariate analysis, there was a statistically significant association between a score of 2 to 3 on the Cognitive Leisure Activity Index and frailty transitions (odds ratio [OR] = 1.93, 95% CI 0.03 to 1.29, *p* = .04), while all other covariates were not significantly different across the three groups. After adjusting for covariates, participants with more cognitive leisure activities had a higher risk of frailty improvement than those without cognitive leisure activities (odds ratio [OR] = 1.99, 95% CI 1.05 to 3.76, *p* = .04).

**Conclusions:**

Cognitive leisure activities were positively associated with the risk of frailty improvement in older adults, mainly when participating in multiple such activities. Older adults may be encouraged to participate in a wide variety of cognitive leisure activities to promote healthy aging.

**Supplementary Information:**

The online version contains supplementary material available at 10.1186/s12889-024-18889-w.

## Introduction

With the growth in population aging, the health of older adults is attracting increased attention. Frailty is one of the most serious forms of population aging since it is an extreme outcome of the normal aging process [1]. Frailty is an age-related clinical condition defined by a deterioration in multiple physiological functions and an age-related loss of physiological reserves, resulting in increased sensitivity to stressors [2, 3]. Frail older adults are more susceptible to health issues such as decreased mobility, falls, delirium, hospitalization and mortality [2–5].

Frailty is a major contributor to increasing health-care expenses [6] for older adults. A cross-sectional study showed that the overall prevalence of frailty in China is 9.9% [7], and this figure is expected to rise in the future. Therefore, preventing and slowing the growth of frailty is a critical subject in the advancement of modern geriatrics [3].

A thorough knowledge of frailty risks and possible protective factors is of great importance for developing strategies to prevent and slow the progression of frailty. In recent years, many researchers have investigated risks that might trigger frailty using various methods. Previous studies have shown that frailty risk factors include sex, age, education, living alone, multiple chronic diseases, low levels of physical activity and sedentary lifestyle, loneliness, depression, and cognitive impairment [7–11].

Cognitive leisure activities refer to events that individuals engage in for pleasure and well-being in addition to their daily lives [12]. Such activities, for example, surfing the internet, watching TV, reading books or newspapers, and playing mahjong, cards and chess, are modifiable lifestyle factors that can stimulate cognitive and intellectual development [13]. Leisure events, particularly for retired older adults, can function as an important part of their lives. Previous research has found that cognitive pastimes can improve older adults’ health in many ways. Playing cards or mahjong, reading newspapers or books, watching television or listening to the radio, and having daily internet access, for instance, can delay cognitive decline, minimize the risk of cognitive impairment, and improve cognitive performance [14–18]. In addition, they are good for avoiding dementia and slowing its progression [19, 20]. Such entertainment can also have an impact on life satisfaction, happiness, depression and stress levels [21], and the frequency of participation is strongly associated with cognitive function and mental health [22, 23]. In terms of mortality, some studies have shown that cognitive activity is related to a lower risk of all-cause mortality in older groups [24]. One particular study shows that regular intellectual activity can lessen the risk of debilitation [25], and the intellectual activities in this study are conceptually, rolewise, and compositionally comparable to cognitive leisure activity. However, the relationship between cognitive leisure activities and frailty transitions has not been adequately studied and reported.

To date, few existing studies have addressed the effect of cognitive leisure activities on frailty regression. Thus, this study was based on data from the CHARLS longitudinal study to investigate the relationship between cognitive leisure events and frailty transitions.

## Methods

### Data collection and population

The data analyzed in this study are from the China Health and Retirement Longitudinal Study (CHARLS) [26]. CHARLS is a longitudinal survey of inhabitants in mainland China aged 45 and older that provides information such as socioeconomic position and health status. It uses a stratified multistage PPS random sample approach and covers 150 districts and 450 villages/urban communities, involving 17,708 people in 10,257 households. To date, four waves of surveys have been conducted in 2011, 2013, 2015 and 2018. Details of the surveys are available in its report [26] and on the official CHARLS website (http://www.charls.pku.edu.cn/en). All surveys were ethically approved by the Institutional Review Board of Peking University.

**Fig. 1:**
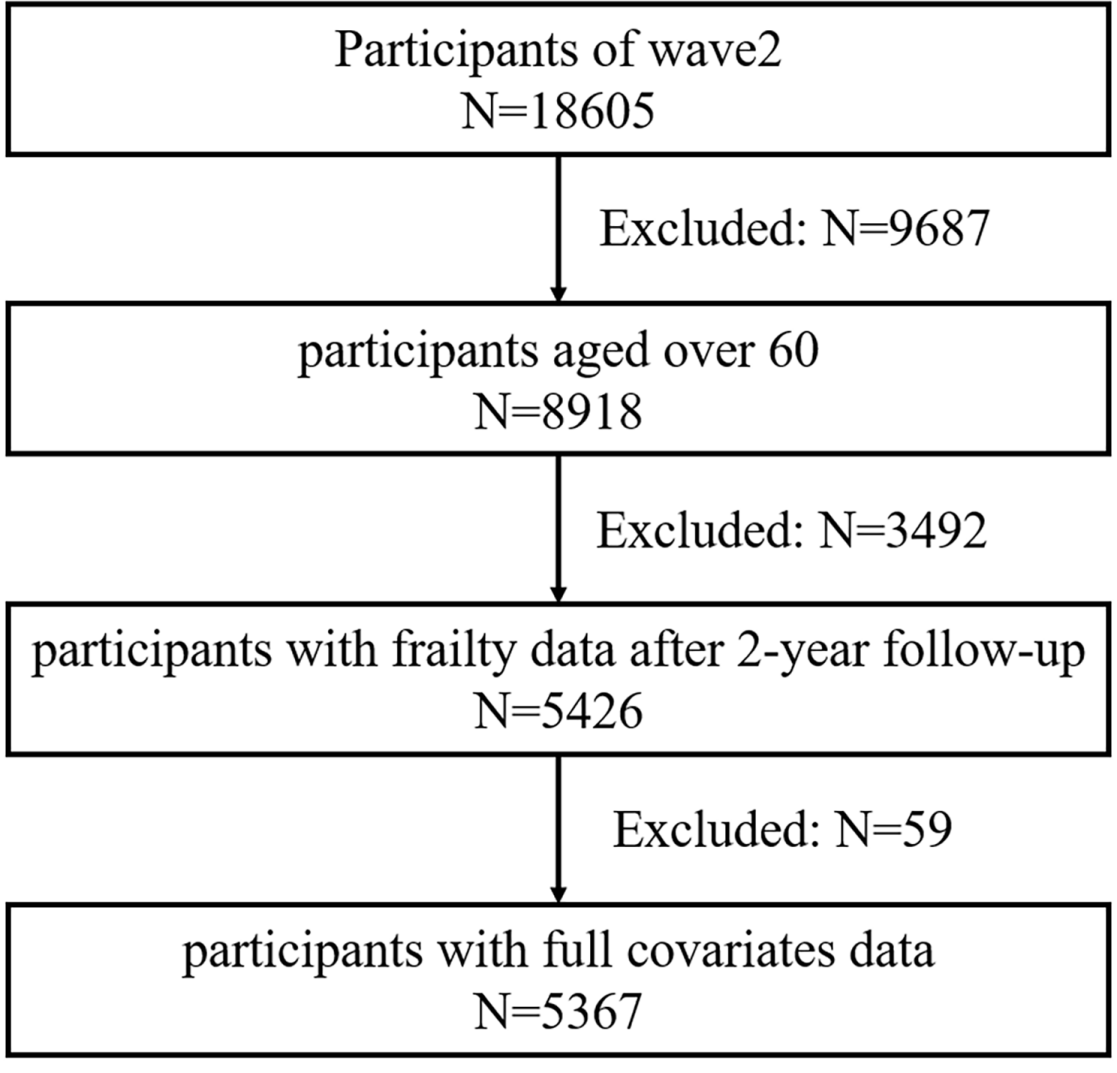
Flowchart of participants selection and exclusion

Because the most recent survey in 2018 lacks part of the data necessary to quantify frailty in older adults, three waves of data from 2011 to 2015 were used for the current study. The 2nd wave of the CHARLS in 2013 was set as a baseline, and data for a total of 18,605 participants were obtained. Since the subjects of this study were older adults, we limited the age of participants to 60 years and above. In 2013, each participant’s cognitive leisure engagement, frailty status, and other relevant sociodemographic and health characteristics were gathered. After 2 years of follow-up, the frailty status of each participant was determined in the 3rd wave of the CHARLS in 2015. The first wave of CHARLS data in 2011 was also included in this study due to the requirement of calculating certain variables. The dummy variable approach was used to assign missing samples of variables with more than 1% missing values, whereas missing samples of variables with fewer than 1% missing values were omitted. The details of data selection and exclusion are presented in Fig. 1. The total number of participants in the 2nd wave of the CHARLS was 18,605. The sample was first screened for age greater than or equal to 60 years, excluding 9687 and leaving 8918. Then, the samples were screened based on the completeness of the data. According to the completeness of the frailty data after the 2-year follow-up, 3492 samples were excluded, leaving 5426 samples. Finally, the sample with complete covariate data was screened, excluding 59 individuals and leaving 5367 individuals for the follow-up study.

### Measurement of cognitive leisure activity participation

The engagement of older adults in cognitive leisure activities was the independent variable in this study, and we conducted research based on the CHARLS. The 2nd wave of the CHARLS in 2013 asked participants if they had engaged in any of the listed social activities in the previous month; the second item on that list, “Played Mahjong, played chess, played cards, or went to community club,” the ninth item “Stock investment,” and the tenth item “Used the internet” were all cognitive leisure activities. Due to the limitations of the CHARLS database and its survey content, only these three cognitive leisure activities were considered in this study. According to the participation status, each cognitive leisure activity was rated as not participating (score = 0) or participating (score = 1). To provide a comprehensive measure of older adults’ participation, we aggregated participation in the three cognitive leisure activities into the Cognitive Leisure Activity Index (CLAI) [24], adding 1 point for each cognitive leisure activity participated in, with a total score ranging from 0 to 3. Since the number of participants with scores of 3 was insufficient for conducting a statistical analysis, the participants with scores of 3 were combined with those with scores of 2 into a 2 ~ 3 score group. Therefore, the cognitive leisure activity index is a trichotomous variable with scores of 0 (no cognitive leisure activity), 1 (less cognitive leisure activity), and 2 to 3 (more cognitive leisure activity).

### Frailty and frailty transitions

The outcomes of the study were frailty and frailty transitions. The FRAIL Scale is used to assess frailty and consists of five components: fatigue, resistance, ambulation, illnesses and loss of weight [27]. The following frailty criteria were used in this study:

Fatigue: Fatigue refers to self-perceived tiredness over a period. In our study, fatigue was measured by two questions from the Center for Epidemiological Studies Depression Scale (CESD). For the questions “I felt everything I did was an effort” and “I could not get ‘going’”, if the participant answered “Occasionally or a moderate amount of the time (3–4 days)” or “Most or all of the time (5–7 days)” in either question, then the participant met the criteria for fatigue and scored one point.

Resistance: Resistance refers to the loss of endurance, which is evidenced by the difficulty in climbing one flight of stairs without resting midway and the help of aids or others. In our study, we asked participants whether they had difficulty climbing several flights of stairs without resting, and if they answered “Yes, I have difficulty and need help” or “I cannot do it”, then the participant met the criteria for resistance and scored one point.

Ambulation: Ambulation refers to a decrease in free movement ability, which is manifested by difficulty walking 100 m without the help of aids or others. In our study, we asked participants if they had difficulty walking 100 m, and if they answered “Yes, I have difficulty and need help” or “I cannot do it”, then the participant met the criteria for ambulation and scored one point.

Illnesses: illnesses reflect the participant’s disease status, which is defined as the presence of five or more diseases, such as hypertension, diabetes, acute heart attack, stroke, angina pectoris, congestive heart failure, asthma, arthritis, chronic lung disease, kidney disease, and malignancy (except microscopic skin cancer), i.e., the coexistence of multiple diseases. In our study, we asked participants about their disease status, and if the presence of 5 or more diseases was reported, then the participant met the criteria for illnesses and scored one point.

Loss of Weight: Loss of weight refers to a weight loss of more than 5% in the last 1 year or less. In our study, this evaluating indicator was judged by participants’ self-report or measurement of weight or body mass index (BMI). If participants answered “Yes, I only lost weight” when asked if they gained or lost 5 or more kilograms in the last year (excluding pregnancy) or if the difference between the weight measurements taken during the adjacent waves of CHARLS showed a weight loss of more than 5% or a BMI was less than 18.5 kg/m^2^, then the participant met the criteria for loss of weight and scored one point.

Each of these five evaluating indicators is worth 1 point, for a total of five points. A score of 0 indicates robustness, 1 to 2 indicates prefrailty, and 3 and above indicates frailty. Prefrailty and frailty were both classified as frailty states, thus representing the polar opposite of robustness.

Using the FRAIL Scale, participants’ frailty status could be measured individually for 2013 and 2015. The transformation in frailty status from 2013 to 2015 during these two waves of the CHARLS is our dependent variable. The difference in frailty between these two waves of the CHARLS may be used to evaluate transitions in frailty, which can be classified as improvement (difference < 0), staying the same (difference = 0) and deterioration (difference > 0). For the purpose of subsequent statistical analysis, we assigned values to the cases of frailty transitions as 0 = worsening, 1 = staying the same, and 2 = improving.

### Covariates

Based on previous research, several sociodemographic and health data were included in the study as covariates. Sociodemographic covariates included age, sex (female = 0, male = 1), educational attainment (no formal education (illiterate) = 0, did not finish primary school but capable of reading and/or writing or Sishu/home school = 1, elementary school = 2, middle school and above = 3), marital status (no spouse = 0, spouse = 1), region of residence (rural = 0, urban = 1), and health insurance coverage (whether or not enrolled in any of the health insurance, no health insurance = 0, enrolled in health insurance = 1). Health science covariates included smoking (not smoking = 0, quit smoking = 1, still smoking = 2) and alcohol use (not drinking = 0, rarely drinking = 1, regularly drinking = 2). It is necessary to clarify that the vast majority of participants (5328/5367, 99.3%) were able to take care of themselves, and that the participants’ ability to perform activities of daily living did not prevent them from participating in cognitive leisure activities. Therefore, ADL was not included in the covariates.

### Statistical analysis

Descriptive statistics were used to present the data. The sample was separated into three groups, and an equilibrium comparison was performed since the exposure factor (cognitive leisure activity index) was an ordinal categorical variable. For continuous variables, the Kolmogorov‒Smirnov test was used to determine the normality of the data. Normally distributed data are expressed as the mean (standard deviation), and skewed data are described as the median (quartiles). ANOVA and the Kruskal‒Wallis test were used for comparisons between three groups of normally and skewed continuous variables, respectively. To depict the changes in frailty distribution and composition, bar charts and pie charts were employed. The changes in frailty distribution and the composition of the frailty state transition were visualized using bar charts and pie charts, respectively, and the characteristics of the frailty state transition between the two waves were displayed using a Sankey diagram (https://www.rawgraphs.io/).

One-factor ordered logistic regression analysis and multifactor ordered logistic regression analysis [28] were used to assess the association between variables and frailty transitions. For the univariate analysis, each baseline characteristic was used as an independent variable to assess its association with the dependent variable. For multifactorial analysis, statistically significant covariates in the equilibrium comparison were considered potential confounders and included in the multifactorial ordered logistic regression analysis. The regression analysis reported the odds ratio (OR) and 95% confidence interval (CI). All statistical analyses were performed using IBM SPSS Statistics 26.

## Results

### Frailty transitions

**Fig. 2:**
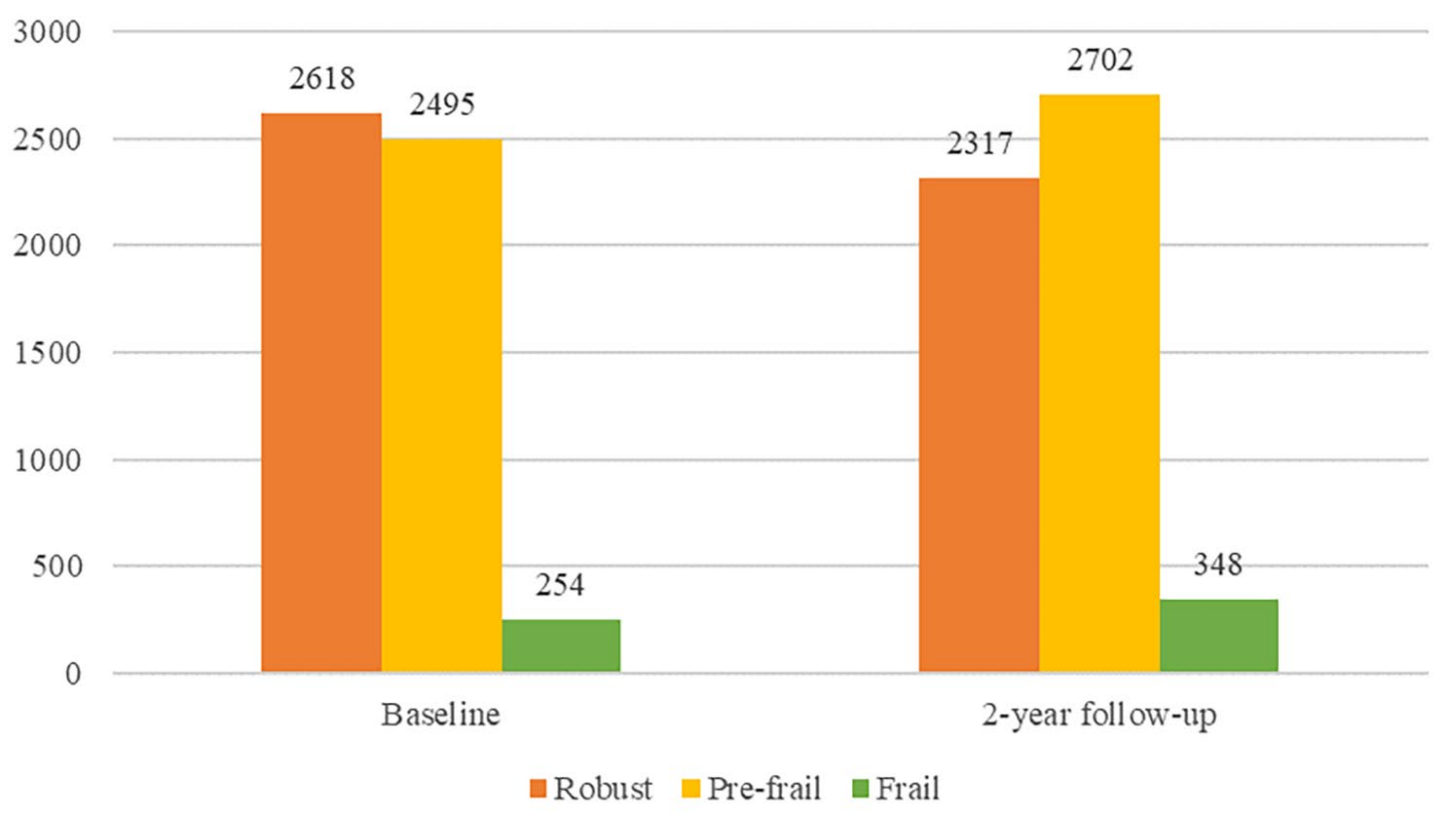
Distribution of frailty at baseline and after 2 years of follow-up

**Fig. 3:**
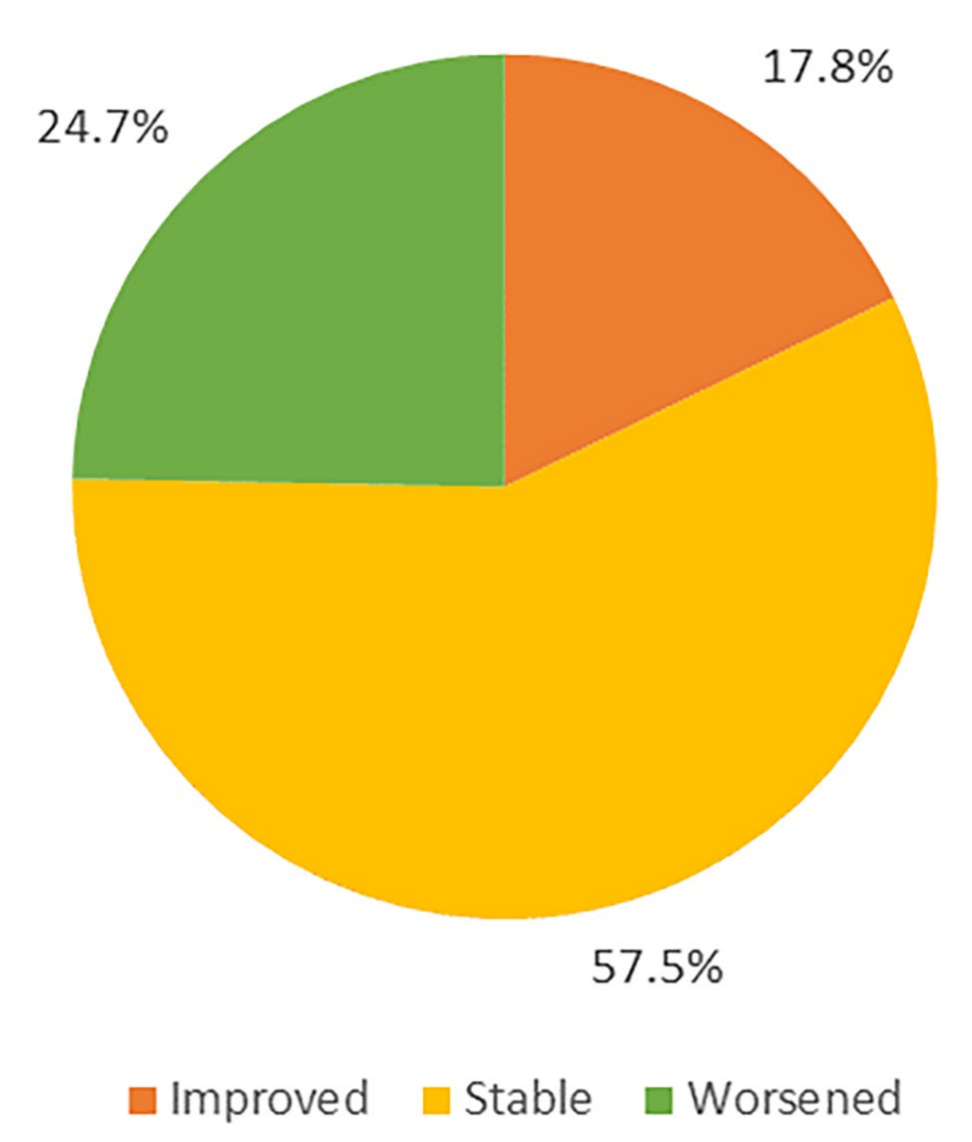
The composition of frailty transitions

A total of 5367 participants were included in this study. As indicated in Fig. 2, At baseline, 2618 (48.8%) were robust, 2495 (46.5%) were prefrail, and 254 (4.7%) were frail. After two years, 2317 (43.2%) were robust, while 2702 (50.3%) and 348 (6.5%) were in the prefrailty and frailty states, respectively. As indicated in Fig. 3, after two years of follow-up, the number of robust participants decreased, while the number of prefrail and frail participants increased, with the rates of improvement and deterioration in frailty status of the population being 17.8% (957/5367) and 24.7% (1326/5367), respectively. Overall, the status of more than half of the participants remained the same.

**Fig. 4:**
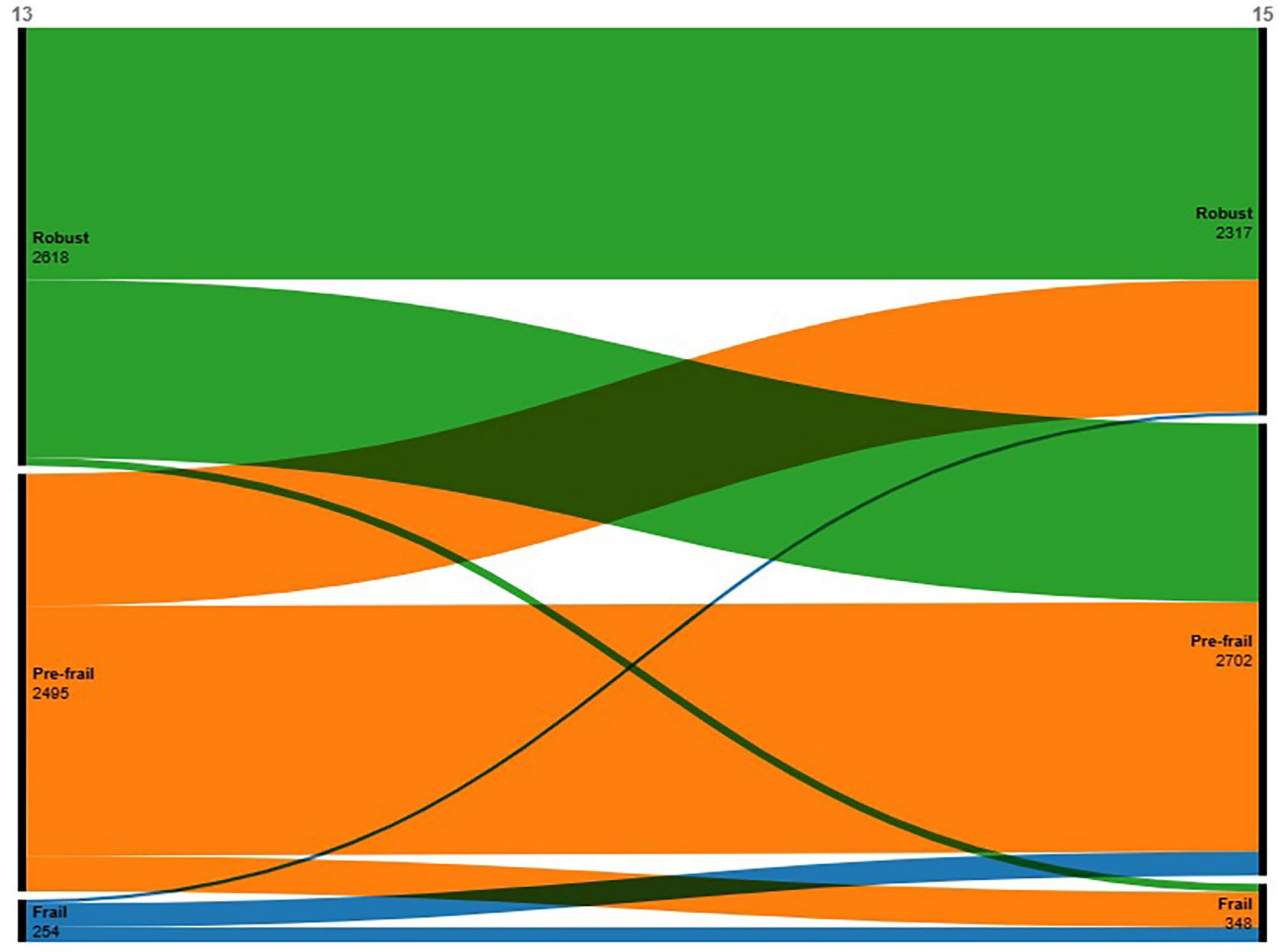
Sankey diagram of the transition in frailty status

For the 2618 participants who were robust at baseline, 40.7% (1066/2618) and 1.8% (48/2618) transformed to prefrail and frail states, respectively. A total of 8.5% (212/2495) of the 2495 prefrail older adults advanced to frailty, whereas 31.7% (791/2495) reverted to a healthy condition. In addition, 56.7% (144/254) and 8.7% (22/254) of the 254 frail older adults recovered to a prefrail and healthy condition, respectively. The above is shown in Fig. 4.

### Descriptive statistics

The sociodemographic and health characteristics of the participants are shown in Table 1. In total, 5367 participants were ultimately recruited into the study, among which 2611 (48.6%) people were female and 2756 (51.4%) were male. The mean age of the participants was 66 (62–71) years. A total of 1802 (33.6%) cases were illiterate, and 1173 (21.9%) respondents were elementary school dropouts or Sishu/home school students. There were 1357 (25.3%) individuals who had completed primary school. The remaining 1035 (19.3%) individuals had a junior high school education or above. The majority of respondents (4412/5367, 82.2%) were married, while the remaining participants (955/5367, 17.8%) were single. Those who reside in rural regions (3455/5367, 64.4%) outnumber those who live in towns (1912/5367, 35.6%). Almost everyone (5211/5367, 97.1%) had medical insurance, with only 156 (2.9%) instances being uninsured. Most residents (2887/5367, 70.7%) did not smoke, while 435 (10.6%) and 763 (18.7%) had quit smoking or continued smoking, respectively. A total of 3571 (66.5%) respondents did not consume alcohol, 382 (7.1%) participants rarely drank alcohol, and 1414 (26.3%) respondents drank alcohol often. The majority of the participants were male, were illiterate, had a spouse, lived in rural areas, had health insurance, did not smoke, and did not drink alcohol.

In terms of cognitive leisure activities, 19.3% (1036/5367) of participants engaged in mahjong, chess, or card games, 0.4% (19/5367) traded stocks, and 1.5% (78/5367) browsed the internet. In terms of number, 79.7% (4276/5367) of participants said they had no cognitive leisure activities, 19.6% (1054/5367) said they had one, and 0.7% (37/5367) said they had two or three.

As shown in Table 1, groups were formed based on the Cognitive Leisure Activity Index scores, and possible confounders were identified using an equilibrium comparison. Individuals with higher scores on the Cognitive Leisure Activity Index were mostly younger participants, male, with junior high school education and above, with spouses, living in towns, having medical insurance, nonsmokers, and nondrinkers. All covariates were significantly different among the three groups and were all potential confounding variables for the cognitive leisure activity index.

**Table 1:** Table of sociodemographic and health characteristics of the old adults

Variable	Total	Cognitive leisure activity index score				
		0	1	2 ~ 3	H / χ2	*P*-value
Total	5367	4276	1054	37		
Age, median (IQR)	66(62–71)	66(62–71)	65(62–70)	63(61–67)	18.7(2)	**< 0.001**
Gender, n(%)					126.6(2)	**< 0.001**
Female*	2611(48.6)	2246(52.5)	352(33.4)	13(35.1)		
Male	2756(51.4)	2030(47.5)	702(66.6)	24(64.9)		
Education attainment, n(%)					356.1(6)	**< 0.001**
Illiteracy*	1802(33.6)	1638(38.3)	164(15.6)	0(0.0)		
dropout or sishu	1173(21.9)	943(22.1)	230(21.8)	0(0.0)		
Primary school	1357(25.3)	1026(24.0)	324(30.7)	7(18.9)		
Middle school or above	1035(19.3)	669(15.6)	336(31.9)	30(81.1)		
Marital status, n(%)					8.2(2)	**0.02**
No*	955(17.8)	793(18.5)	156(14.8)	6(16.2)		
Yes	4412(82.2)	3483(81.5)	898(85.2)	31(83.8)		
Region, n(%)					108.9(2)	**< 0.001**
Rural*	3455(64.4)	2871(67.1)	582(55.2)	2(5.4)		
Urban	1912(35.6)	1405(32.9)	472(44.8)	35(94.6)		
Medical insurance, n(%)					9.2(2)	**0.01**
No*	156(2.9)	139(3.3)	17(1.6)	0(0.0)		
Yes	5211(97.1)	4137(96.7)	1037(98.4)	37(100.0)		
Smoking, n(%)					75.0(4)	**< 0.001**
Non-smoker*	2887(70.7)	2472(73.6)	398(56.9)	17(65.4)		
Ex-smoker	435(10.6)	317(9.4)	114(16.3)	4(15.4)		
Smoker	763(18.7)	571(17.0)	187(26.8)	5(19.2)		
Drinking, n(%)					93.8(4)	**< 0.001**
No*	3571(66.5)	2961(69.2)	594(56.4)	16(43.2)		
Rarely	382(7.1)	281(6.6)	90(8.5)	11(29.7)		
Often	1414(26.3)	1034(24.2)	370(35.1)	10(27.0)		

* control group

### Cognitive leisure activities and the risk of frailty transitions

Table 2 shows the findings of unadjusted and adjusted ordered logistic regression analysis with frailty transition as the dependent variable (with robustness as the control) and cognitive leisure activity index scores as the independent variable. Univariate ordered logistic analysis demonstrated that higher Cognitive Leisure Activity Index scores in older adults were linked with frailty improvement (odds ratio [OR] = 1.93, 95% CI 0.03 to 1.29, *p* = .04). Although there was no statistically significant association between a cognitive activity index score of 1 and frailty transitions, the group with scores of 2 to 3 significantly differed. Specifically, older adults who participated in 2–3 cognitive leisure activities had 1.93 times greater risk of frailty tending to improve compared to those who did not participate. Covariates such as age, sex, educational level, area, health insurance coverage, smoking, and alcohol usage were all adjusted in multifactorial ordered logistic regression. The findings revealed that engagement in greater cognitive leisure activities was associated with frailty transitions and was a promoting factor for frailty improvement (odds ratio [OR] = 1.99, 95% CI 1.05 to 3.76, *p* = .04). Similar to the results obtained during the univariate ordered logistic regression analysis, statistical significance between the cognitive activity index and frailty transitions existed only when the cognitive activity index score was 2 to 3. Older adults who participated in 2–3 cognitive leisure activities had an approximately 1.99 times higher risk of improving one level of frailty toward improvement than those who did not participate in cognitive leisure activities.

In addition, in univariate ordered logistic regression analysis, there was no statistically significant relationship between all other covariates and frailty transitions. After adjustment, only gender was statistically significant in relation to frailty transitions (odds ratio [OR] = 1.18, 95% CI 1.01–1.38, *p* = .04), and men were at greater risk of frailty improvement than women.

**Table 2:** Table of the results of the ordered logistic regression analysis with the dependent variable of frailty transitions

Variable	Unadjusted			Adjusted		
	OR	95% CI	*P* value	OR	95% CI	*P* value
cognitive leisure activity index scores						
0*						
1	1.01	-0.12 to 0.14	0.85	1.02	0.89 to 1.17	0.75
2 ~ 3	1.93	0.03 to 1.29	**0.04**	1.99	1.05 to 3.76	**0.04**
Age	0.99	-0.01 to 0.00	0.21	0.99	0.98 to 1.00	0.07
Gender, n(%)						
Female*						
Male	1.03	-0.07 to 0.14	0.56	1.18	1.01 to 1.38	**0.04**
Education attainment, n(%)						
Illiteracy*						
dropout or sishu	1.01	-0.13 to 0.15	0.90	0.98	0.85 to 1.14	0.81
Primary school	0.99	-0.15 to 0.12	0.83	0.95	0.82 to 1.11	0.53
Middle school or above	0.98	-0.17 to 0.13	0.78	0.92	0.78 to 1.09	0.32
Marital status, n(%)						
No*						
Yes	0.92	-0.22 to 0.06	0.24	0.89	0.77 to 1.03	0.11
Region, n(%)						
Rural*						
Urban	1.07	-0.04 to 0.18	0.21	1.08	0.96 to 1.21	0.19
Medical insurance, n(%)						
No*						
Yes	0.79	-0.54 to 0.08	0.14	0.80	0.59 to 1.09	0.16
Smoking, n(%)						
Non-smoker*						
Ex-smoker	0.93	-0.27 to 0.12	0.47	0.88	0.71 to 1.09	0.24
Smoker	1.02	-0.14 to 0.17	0.83	0.95	0.79 to 1.15	0.61
Drinking, n(%)						
No*						
Rarely	0.86	-0.36 to 0.05	0.14	0.82	0.67 to 1.02	0.07
Often	0.94	-0.18 to 0.06	0.34	0.92	0.80 to 1.05	0.20

* control group

## Discussion

### Principal results

To our knowledge, this is the first study designed to analyze the relationship between cognitive leisure activities, including playing mahjong and cards, stock investment and internet surfing, and frailty transitions. After a 2-year follow-up, we found that cognitive leisure activities were associated with frailty transitions in Chinese senior citizens, with the number of activities playing an essential role. Although there was no statistically significant relationship between participating in a single cognitive leisure activity and frailty transitions, participation in two to three cognitive leisure activities was associated with a 0.99 higher risk of frailty improvement. The present study suggests that participation in various cognitive leisure activities is a promoting factor for frailty improvement and influences frailty development and improvement.

A significant relationship between cognitive leisure activities and frailty transitions was identified through regression analysis. Similarly, Huang Y et al. [25] noted that regular intellectual activities were beneficial to decrease the risk of frailty, while Abe T et al. [29] found an ameliorating effect of intellectual activity and social participation on frailty status through a cohort study. Although the focus varies, cognitive leisure activities have a high degree of overlap with intellectual activities and social participation in terms of composition. The effect of cognitive leisure activities on cognitive status has been validated in many previous studies in a variety of ways. Litwin H et al. [30] reported that cognitively stimulating leisure activities (word or number games) are a potential cause of delayed or reduced late cognitive decline. Mao C et al. [19] noted that specific leisure activities, including watching television or listening to the radio, reading books or newspapers, and playing cards or mahjong, may reduce the risk of cognitive impairment in older adults. Iizuka A et al. [12] and Fallahpour M et al. [31] both conducted a systematic evaluation of the relationship between leisure activities and cognitive ability and found that participation in leisure activities improved cognitive function and had a preventive effect on dementia in older adults. Notwithstanding variances in study techniques, assessment standards, and other factors, these studies are congruent in their understanding of the relationship between cognitive leisure activities and cognitive health, indicating that these findings are generalizable. The negative correlation of cognitive leisure activities with the likelihood of frailty deterioration in older adults further confirmed the inverse link between cognitive leisure activities and cognitive impairment in our study. The link between cognitive impairment and frailty has long been known. According to Yuan Y et al., cognitive impairment is significantly linked to frailty and is a risk factor for frailty [32]. In turn, frailty is also significantly associated with lower cognitive ability [33], and cognitive impairment is much more likely to occur in frail than in normal individuals [34]. Thus, cognitive impairment may be a mediating variable in the influence of cognitive leisure activities on frailty transitions, i.e., cognitive leisure activities may influence the risk of frailty transitions in older adults in part through their negative association with cognitive impairment.

For the three cognitive leisure activities researched in this study, there is also relevant literature support for their independent relationship with cognitive health. It has been found that playing mahjong or cards could maintain and improve cognitive function in older adults [22] and lower the risk of dementia [19]. Moderate internet access is not only effective in improving the health of older adults [35] but also in improving cognitive function and slowing cognitive decline [36, 37], possibly through the effect on pallidum volume [38]. Although it has not been well investigated, speculation nonetheless qualifies as a cognitive leisure activity due to the method in which it is carried out. There are several more activities that may be categorized as cognitive leisure activities in addition to these three, but they were the only options for cognitive leisure activities due to database restrictions. A diverse range of cognitive recreational activities can more accurately reflect the function of cognitive recreational activities. Research on diverse cognitive leisure activities is moving in a mixed direction right now, which is a desirable path for future study.

Additionally, our multifactorial regression model revealed that female sex was a risk factor for frailty deterioration, which is in line with earlier research [39]. Although the effect is small, gender is indeed associated with frailty, and its explanations are multifaceted. According to some research, this might be due to sex disparities in literacy among older adults [24].

Our results derived from longitudinal research also show that frailty is a dynamic process that is both progressive and reversible. Although more than half of the participants remained in the same state (3084/5367, 57.5%) and even a number advanced to a more severe state of frailty (1326/5367, 24.7%), there were still a small part of participants who improved (957/5367, 17.8%), suggesting that appropriate measures could be taken to intervene in the onset and progression of frailty.

China is one of the nations that is aging the fastest and has a sizable senior population [40]. As China’s population ages, many difficulties associated with aging become increasingly visible. In terms of health care, the enormous health-care expenses associated with aging place a substantial strain on China’s health-care and public-health systems, where frailty plays a large role. As a result, we must vigorously promote active and healthy aging. Lifestyle modifications are critical, and the favorable impacts of leisure activities on health and frailty have been well documented. We may encourage older adults to participate in more cognitive leisure activities depending on their preferences and aim toward active aging by delaying or suppressing frailty progression.

### Cognitive leisure activities

Since there is no standardized scale for cognitive leisure activity, this study defined the Cognitive Leisure Activity Index (CLAI) based on the literature and CHARLS. Therefore, it is necessary to acknowledge that the CLAI is not a structured evaluation scale with established validity and reliability. It is expected that relevant standardized assessment scales will be available for researchers to use in the future.

Only three cognitive leisure activities—playing mahjong or cards, investing in stocks, and using the internet—were covered in this study because of the limits of the CHARLS. This limited the study’s ability to capture all cognitive leisure activities, and there were many other cognitive leisure activities that could not be included in the study.

Regarding these three cognitive leisure activities, there may be a correlation between Internet use and stock investment. To rule out this concern, the data was statistically analyzed and it was found that among the individuals involved in stock investments, 15 (79.0%) of them used the Internet, while 4 (21.0%) of them did not use the Internet. This statistically indicates that the use of the internet is not necessarily a necessary condition for stock investment, ruling out its influence on the results of the study.

Frequency of participation in cognitive leisure activities is an index worth considering, which may improve the accuracy of the study’s results. Due to the dimensional inconsistency among three cognitive leisure activities, however, this study did not perform additional research on how frequently people engage in cognitive leisure activities. Certainly, it can be considered that the amount of participation in cognitive leisure activities can, to some extent, reflect the frequency of participation in cognitive leisure activities.

### Strengths and limitations

The link between cognitive leisure activities, including similar intellectual activities, and frailty has been observed several times, but its impact on frailty transitions is still unclear. To our knowledge, this is the first study to demonstrate a relationship between cognitive leisure activities and frailty transitions, and the findings might provide evidence and methods to enhance frailty management and progression control. In a broad sense, cognitive leisure activities also belong to a type of lifestyle. Currently, lifestyle adjustment has become an important measure to achieve healthy aging. The findings of this study complement and improve the theories related to healthy aging and can provide more guidance for the realization of healthy aging.

The study does, however, have flaws. The samples for the study were all drawn from the CHARLS, showing that the study used only one database and thus had a somewhat limited scope. Moreover, most of the data came from participants’ responses to questionnaires and self-reports, rather than more accurate laboratory or clinical trials. However, self-reported data are still considered valid and credible by many publishers. In contrast, some of the data derived from the measures (such as height and weight) were recorded with somewhat evident errors, which made data processing more difficult and, to some extent, impacted the accuracy of the variables. There were some samples with some missing data. A portion of the missing data would still inevitably have a negative effect even though the missing values of variables with more than 1% missing data were assigned in this study using the dummy variable method; only samples with missing exposure factors and dependent variables and missing samples of covariates with less than 1% missing data were excluded. These limitations offer fresh perspectives for future research.

## Conclusions

In conclusion, this study of older Chinese adults revealed that cognitive leisure activities, including playing mahjong or cards, stock investment, and using the internet, can slow frailty deterioration and contribute to its improvement and had a more significant impact when engaging in multiple activities. Given this tendency, we should encourage older adults, especially those who are already in a frail state, to engage in more various cognitive and leisure activities in an effort to achieve active aging.

## Electronic supplementary material

Below is the link to the electronic supplementary material.

Supplementary Material 1

## Data Availability

The datasets analyzed during the current study are available on the official CHARLS website (http://charls.pku.edu.cn/en/).
