# Supplementary material for: The effects of cognitive leisure activities on frailty transitions in older adults in China: a CHARLS-Based longitudinal study
Source: BMC Public Health. 2024 May 27;24:1405. doi: 10.1186/s12889-024-18889-w (PMC11129477; doi:10.1186/s12889-024-18889-w)
Supplement: Supplementary file 1 — Supplementary Material 1 [file 12889_2024_18889_MOESM1_ESM.docx]

***Measurement of Cognitive Leisure Activity Participation***

| **Cognitive Leisure Activity** | Have you done any of these activities in the last month? (Code all that apply) | 1. Interacted with friends | / |
| --- | --- | --- | --- |
|  |  | 2. Played Ma-jong, played chess, played cards, or went to community club | 1 |
|  |  | 3. Provided help to family, friends, or neighbors who do not live with you | / |
|  |  | 4. Went to a sport, social, or other kind of club | / |
|  |  | 5. Took part in a community-related organization | / |
|  |  | 6. Done voluntary or charity work | / |
|  |  | 7. Cared for a sick or disabled adult who does not live with you | / |
|  |  | 8. Attended an educational or training course | / |
|  |  | 9. Stock investment | 1 |
|  |  | 10. Used the Internet | 1 |
|  |  | 11. Other | / |
|  |  | 12. None of these | / |

***The FRAIL Scale***

| **fatigue** | I felt everything I did was an effort. | 1. Rarely or none of the time (< 1 day)  2. Some or a little of the time (1-2 days)  3. Occasionally or a moderate amount of the time (3-4 days)  4. Most or all of the time (5-7 days) | 1 |
| --- | --- | --- | --- |
|  | I could not get “going”. | 1. Rarely or none of the time (< 1 day)  2. Some or a little of the time (1-2 days)  3. Occasionally or a moderate amount of the time (3-4 days)  4. Most or all of the time (5-7 days) |  |
| **resistance** | Do you have difficulty with climbing several flights of stairs without resting? | 1. No, I don’t have any difficulty  2. I have difficulty but can still do it  3. Yes, I have difficulty and need help  4. I can not do it | 1 |
| **ambulation** | Do you have difficulty with walking 100 metres? | 1. No, I don’t have any difficulty  2. I have difficulty but can still do it  3. Yes, I have difficulty and need help  4. I can not do it | 1 |
| **illnesses** | Have you been diagnosed with [conditions listed below, read one by one] by a doctor? | 1. Hypertension  2. Dyslipidemia (elevation of low density lipoprotein, triglycerides (TGs), and total cholesterol, or a low high density lipoprotein level)  3. Diabetes or high blood sugar  4. Cancer or malignant tumor (excluding minor skin cancers)  5. Chronic lung diseases, such as chronic bronchitis, emphysema (excluding tumors,  or cancer)  6. Liver disease (except fatty liver, tumors, and cancer)  7. Heart attack, coronary heart disease, angina, congestive heart failure, or other heart problems  8. Stroke  9. Kidney disease (except for tumor or cancer)  10. Stomach or other digestive disease (except for tumor or cancer)  11. Emotional, nervous, or psychiatric problems  12. Memory-related disease  13. Arthritis or rheumatism  14. Asthma | 1 |
| **loss of weight** | Have you gained or lost 5 or more kilograms in the last year? (excluding pregnancy) | 1. Yes, I only gained weight  2. Yes, I only lost weight  3. Yes, I first gained and then lost weight  4. Yes, I first lost and then gained weight  5. No  6. I don’t know | 1 |
|  | whether the difference between the weight measurements taken during the adjacent waves of CHARLS showed a weight loss of more than 5% | |  |
|  | BMI was less than 18.5 kg/m^2^ | |  |
